# Working Memory Load Enhances the Attentional Capture of Low Reward History

**DOI:** 10.3389/fpsyg.2019.02722

**Published:** 2019-12-06

**Authors:** Yujie Wu, Tingni Li, Zhe Qu

**Affiliations:** ^1^Department of Psychology, Sun Yat-sen University, Guangzhou, China; ^2^State Key Laboratory of Cognitive Neuroscience and Learning, IDG/McGovern Institute for Brain Research, Beijing Normal University, Beijing, China

**Keywords:** attentional capture, reward, working memory, cognitive control, load theory

## Abstract

Attention priority of reward history, also called value-driven attentional capture (VDAC), is different from that of saliency or contingency. The magnitude of VDAC was found to be correlated with working memory capacity, but how cognitive control interacts with the attentional allocation of reward association is not clear. Here, we examined whether the distraction by learned color-reward association would change under different working memory load conditions. Participants were first trained with color-reward associations by searching a green/red circle with low/high reward. Then, during the test session, participants needed to search a unique shape while a green/red shape was either presented as a distractor or not shown at all. To manipulate the working memory load in the test, a digital memory task was integrated with the visual search task in half of the trials (memory load condition), but not in the other half (no-load condition). Consistent results were found in two experiments that the magnitude of attentional capture caused by low-value distractors was larger under memory load condition than under no-load condition, while there was no enough evidence supporting the influence of memory load on attentional capture by high-value distractors. These results suggested that working memory load, which occupied part of cognitive resources, reduced the priority of target information and might also modulate the strength of reward association holding in working memory. These findings extend the knowledge regarding the influence of working memory load on attentional capture of reward and suggest that reward-induced distraction is dynamic and could be modulated by cognitive control.

## Introduction

Reward experience is appealing and powerful, which can guide our attention and shape our behavior. As we all know, attention plays an important role in selecting sensory input into awareness. In recent years, an increasing number of studies found that reward history can induce capture of attention (Raymond and O’Brien, 2009; Failing and Theeuwes, 2014), which has challenged the traditional view of dichotomy of attentional selection (Anderson et al., 2011b; Awh et al., 2012): one is top-down or goal-directed attentional selection, which means the stimuli that are related to current goal will get attention (Wolfe et al., 1989); the other one is bottom-up or stimulus-driven attentional capture, which means attention will be captured by a stimulus that is salient on physical properties (e.g., Theeuwes, 1992). So, it is important to explore the cognitive basis of attentional priority of reward history and reveal the interactions between it and other cognitive processes.

The attentional priority of reward-related information was first observed in visual search tasks. First, participants need to search the target of certain colors and get rewards trial by trial in a training session. After that, in the test session, the target changes into a unique shape, and the former target color turns to be one of the distractors in some trials and no reward will be given anymore. As a result, the reaction times (RTs) of subjects get longer when the distractors that used to be associated with reward during training appeared compared to no reward-related distractor, which is called value-driven attentional capture (VDAC, Hickey et al., 2010; Anderson et al., 2011b; Awh et al., 2012; Chelazzi et al., 2013).

Value-driven attentional capture is different from goal-directed attention selection and stimulus-driven attention capture for evidence from three aspects. First, even if the color that is rewarded during training is unique among distractors, the high and low value of color make the effect of VDAC different (Anderson et al., 2011a), which means the capture is not determined by the salient color itself but by the value of reward. Second, the reward-related stimulus has no common with a current target, and its effect is context-dependent. Anderson (2015) found the effect size of VDAC depends on the situation where the association of reward value is built. Third, learning the association between a stimulus and a reward is an indispensable part of VDAC. The repeated occurrence of a target and a reward makes the brain generate a predictive signal for the target when it shows up, which modulates the priority of attention (Sali et al., 2014). Accordingly, reward history seems to attract attention in a direct and special way rather than through a goal-directed or stimulus-driven way. Although their mechanisms are different, saliency and reward can have interactions on attention. For example, Wang et al. (2013) found that VDAC was easier to appear when the reward was associated with color than with shape. However, it remains unclear how VDAC interacts with goal-directed cognitive control.

Working memory acts as an important role in goal-directed attention control (Desimone and Duncan, 1995; Baddeley, 1996). Recent studies revealed that working memory also plays an important role in VDAC. For example, Anderson et al. (2011a) found that people with lower working memory capacity exhibited stronger attentional capture by stimuli with reward history. Another eye movement study (Anderson and Yantis, 2012) further verified a negative correlation between working memory capacity and extent of VDAC. However, these studies did not manipulate the cognitive control to reveal the modulation of working memory on VDAC directly.

Working memory load will occupy cognitive resources and may influence the attentional selection in two contradictory ways. The first way is that, working memory load might impair attentional selection because the processing of distractors cannot be well inhibited by executive control. An fMRI study found that correctly allocating attention to target location needs task-related information with good priority in working memory; high working memory load will impair the priority of target and hence processing of distractor will get enhanced (De Fockert et al., 2001). Specifically, in a flanker task, the distraction is stronger under high working memory load than low working memory load (Lavie et al., 2004). Similarly, in a visual search task, memorizing digits at the same time makes performance more susceptible to irrelevant unique-color stimuli compared to no memory task condition (Lavie and De Fockert, 2005). The second way is that, working memory load might reduce distraction and facilitate allocation of attention to targets. SanMiguel et al. (2008) found that, in a visual classification task, working memory load will reduce the distraction of novel sound both behaviorally and as an index by an attenuation of the late phase of the novelty-P3 EEG signal. So, it is proposed that the effect of working memory load on distraction depends on the nature of the distraction (SanMiguel et al., 2008). Considering the priority of reward history is learning- and context-dependent, which is different from that of saliency, the influence of working memory load on VDAC is worthy to be investigated.

In the current study, we aim to reveal the modulation of working memory load on VDAC directly. Given the previously mentioned two different ways concerning the relationship between working memory and distraction, working memory load may enlarge or reduce VDAC. However, in most studies of VDAC, the reward is associated with visual stimuli that can cause response conflict in visual attention tasks, which is different from totally task-irrelevant auditory distractions used in SamMiguel’s visual classification task (2008). So, here we suppose that the effect of VDAC by visual stimuli would get stronger under working memory load condition. By combining a dual-task paradigm (Pashler, 1994; Lavie and De Fockert, 2005; Muller-Gass and Schröger, 2007) with the value-learning procedure, we manipulated working memory load through a digital memory task and measured VDAC in a visual search task to test the hypothesis.

## Experiment 1

### Materials and Methods

#### Participants

Nineteen undergraduates participated in the formal experiment. One participant did not gaze at the fixation point during the experiment and was rejected from the analysis. Data of eighteen participants were analyzed (nine males and nine females). They were 17–22 years old with a mean age of 19.5 (*SD* = 1.29). All of them had normal or corrected-to-normal visual acuity and had no mental disease history. The experiment was approved by the Ethics Committee of the Department of Psychology, Sun Yat-sen University. Before the experiment, all participants signed informed consent in accordance with the Declaration of Helsinki.

#### Task and Stimuli

To examine the modulation of working memory on the attentional priority of reward history, two variables, working memory load (no load/load) and association between color and reward (no reward/low reward/high reward), were manipulated within subjects. During the training session, the associations were built through visual search and reward feedback trial by trial. In the test session, each participant finished both a single task (with no working memory load) and a dual task (with working memory load).

**FIGURE 1:**
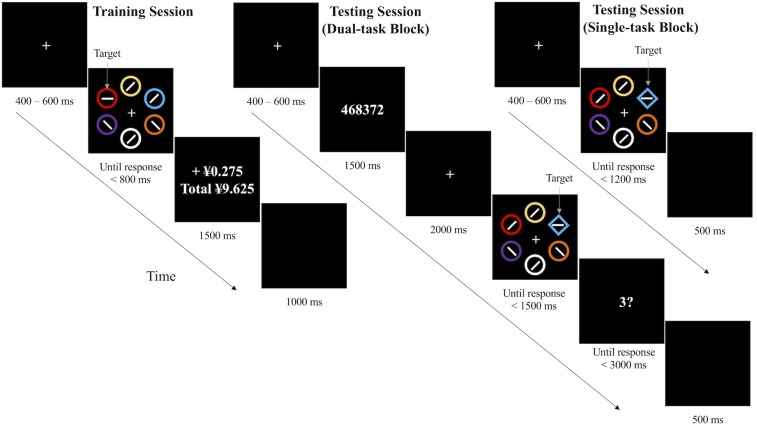
Trial procedures of the training and test sessions.

In the visual search task during the training session, six circles (each 2.3° × 2.3°) with different colors (all possible colors used in training and testing session: red, green, blue, cyan, pink, orange, yellow, white) were presented with an equal distance in an imaginary ring (10° × 10°) (Figure 1). Search targets were red and green circles, and only one of them would appear in each trial. In a red or green circle, there was a horizontal or vertical white bar (1.1°). In circles with other colors, the bars were 45 degrees off-axial directions. Participants were required to judge the orientation of the bar in a red or green circle as soon as possible. The reward of the current trial and total amount of reward appeared after response. There were two kinds of color-reward associations which were balanced between subjects. For half participants, red color was associated with high reward and green color was associated with low reward. For the other half participants, red color was associated with low reward while green color was associated with high reward. In each trial, if the color (red/green) associated with high reward showed up and the bar inside it was correctly responded, then the participant would receive 0.275 yuan with an 80% possibility or 0.055 yuan with a 20% possibility. If the target was the color (green/red) with low reward, then the participant would receive 0.055 yuan with an 80% possibility or 0.275 yuan with a 20% possibility.

In the visual search task during the testing session, five diamonds and one circle or five circles and one diamond composed an imaginary ring in the search display. Participants were asked to judge the orientation of the bar (horizontal or vertical) in a unique shape. Bars in other shapes were 45 degrees off-axial directions. All six shapes had different colors, and in 2/3 trials, one of the distractors was red or green. In the remaining 1/3 trials, which is the no-reward condition, all colors appeared in the training session, but none of them was associated with reward before. Participants were informed that there would be no reward in the test session and the colors were irrelevant anymore.

In working memory task during the test session, for each trial, six numbers which were randomly chosen from 1 to 9 were presented in the center of the screen. No three consecutive numbers were incrementing or decrementing, and all numbers appeared with equal probabilities. The probe number was also randomly chosen from 1 to 9. Participants were asked to report whether the probe number was among the memory array.

#### Procedure

Each participant was tested in a dimly lit room, sitting in front of the screen with a viewing distance of 50 cm. Before training, participants practiced for 24 trials with no reward. There were 240 trials in the training session, and 360 trials in the test session. In the test session, half trials were under single-task condition (i.e., without working memory task), and the other half were under dual-task condition (i.e., with working memory task). The two conditions were divided into two blocks separately, and the order of blocks was balanced between subjects.

In the training session, each trial began with a fixation display for 400, 500, or 600 ms randomly. Then search display appeared until response or for 800 ms. Participants were asked to press key *m* for a vertical bar or press key *z* for a horizontal bar in the target circle as soon as possible. Incorrect responses were followed by a 1000 Hz beep with 100 ms duration. After that, the feedback display showed up for 1500 ms to inform participants of the current reward and their total rewards. The inter-trial interval was 1000 ms.

In the test session, for the dual-task condition, each trial began with a fixation display (400, 500, or 600 ms duration) and then was followed by a digit array for 1500 ms. Participants were told to memorize all numbers in the array and to judge whether the probe number was one of them at the end of the trial. Then after another fixation display for 2000 ms, a search display appeared. Participants needed to press key *m* for a vertical bar or to press key *z* for a horizontal bar in the unique shape as soon as possible. After response or 1500 ms, the probe number appeared, and participants should press *a* for yes and *k* for no within 3000 ms. The inter-trial interval was 500 ms.

For the single-task condition, a search display appeared following an initial random period of fixation (400, 500, or 600 ms) and waited for response until 1200 ms. And the trial ended with an inter-trial interval of 500 ms.

#### Analysis

According to the appearance of reward associated colors, the trials in the test session were classified as three conditions: high reward, low reward, and no reward. Besides, based on the single-task or dual-task condition, trials in the test session were also tagged as no-load and load conditions. A within-subject repeated measures analysis of variance (ANOVA) was used to analyze RTs and error rates. All the statistical tests were conducted by using SPSS (version 22), and the statistical threshold was set at 0.05. *Post hoc* analysis was performed using Fisher’s Least Square Difference (LSD) test (Milliken and Johnson, 1984).

### Results

#### Value-Driven Attentional Capture

In the training session, RTs of high-reward and low-reward conditions were comparable [*t*(17) = −0.72, *p* = 0.479]. We divided the training session into two phases and conducted a two-way repeated measures ANOVA to reveal the training effect. A significant main effect of training was found [*F*(1, 17) = 17.72, *MS*_e_ = 439.59, *p* = 0.001, ${\text{η}}_{\text{p}}^{2}$ = 0.51], with shorter RTs in the second phase (527.21 ± 40.00 ms, *M* ± *SD*) compared to the first phase (548.02 ± 31.48 ms). However, there was no significant reward effect [*F*(1, 17) = 0.90, *MS*_e_ = 295.00, *p* = 0.356, ${\text{η}}_{\text{p}}^{2}$ = 0.05] or interaction between reward and training [*F*(1, 17) = 0.02, *MS*_e_ = 6.47, *p* = 0.882, ${\text{η}}_{\text{p}}^{2}$ = 0.00]. So, participants responded faster with more training and two target colors had roughly equal attentional priority during training.

Reaction times and error rates of visual search task in the test session are shown in Table 1. A repeated measures ANOVA on RTs showed a significant memory load effect [*F*(1, 17) = 89.82, *MS*_e_ = 8307.20, *p* < 0.001, ${\text{η}}_{\text{p}}^{2}$ = 0.84], a significant reward effect [*F*(2, 34) = 8.53, *MS*_e_ = 701.62, *p* = 0.001, ${\text{η}}_{\text{p}}^{2}$ = 0.33], and a significant interaction between them [*F*(2, 32) = 5.63, *MS*_e_ = 401.80, *p* = 0.008, ${\text{η}}_{\text{p}}^{2}$ = 0.25]. Then we analyzed the reward effects in single-task and dual-task conditions separately. Consistent with previous studies on VDAC, in the single-task condition we found a significant reward effect [*F*(2, 34) = 6.22, *MS*_e_ = 433.78, *p* = 0.005, ${\text{η}}_{\text{p}}^{2}$ = 0.27] and a linear trend on three reward value levels (no vs. low vs. high) [*F*(1,17) = 11.43, *MS*_e_ = 457.22, *p* = 0.004, ${\text{η}}_{\text{p}}^{2}$ = 0.40], which indicated that attentional capture cannot be merely explained by the selection history in training session. *Post hoc* comparisons showed significant longer RTs in low- and high-reward conditions compared to no-reward condition (Fisher’s LSD: *p* = 0.012 and *p* = 0.004, respectively). As for the dual-task condition, the reward effect was also significant [*F*(2, 34) = 8.28, *MS*_e_ = 669.64, *p* = 0.001,${\text{η}}_{\text{p}}^{2}$ = 0.33]. *Post hoc* comparisons showed significant longer RTs in low-reward condition compared to no-reward and high-reward conditions (Fisher’s LSD: *p* < 0.001 and *p* = 0.010, respectively), but there was no significant RT difference between no-reward and high-reward conditions (Fisher’s LSD: *p* = 0.288).

**TABLE 1:** Descriptive statistics of Experiment 1 (*N* = 18).

	**Reaction time (standard deviation) (in millisecond)**		**Error rate (standard deviation) (in percent)**	
**Distractor type**	**No load**	**Load**	**No load**	**Load**
No reward	646.64 (88.16)	810.69 (92.47)	11.60 (5.79)	4.37 (5.10)
Low reward	662.43 (101.68)	845.10 (101.47)	11.39 (7.61)	7.84 (6.73)
High reward	670.74 (97.16)	821.89 (108.72)	13.12 (6.40)	5.81 (4.26)

A repeated measures ANOVA on error rates showed a significant main effect of working memory load [*F*(1,17) = 18.51, *MS*_e_ = 52.94, *p* < 0.001, ${\text{η}}_{\text{p}}^{2}$ = 0.52]. No significant main effect of reward [*F*(2,34) = 29.29, *MS*_e_ = 18.06, *p* = 0.212, ${\text{η}}_{\text{p}}^{2}$ = 0.09] or interaction between reward and memory load [*F*(2,32) = 3.18, *MS*_e_ = 13.13, *p* = 0.054 < 0.01, ${\text{η}}_{\text{p}}^{2}$ = 0.16] was found. Different with a previous study (Lavie and De Fockert, 2005), we found error rates was higher when there was no memory load compared to when there was memory load, suggesting that participants in the current experiment was more careful in the dual task. Since neither reward effect nor interaction was significant for error rates, there was no trade-off between error rates and RTs.

#### The Interaction Between Reward and Working Memory Load

**FIGURE 2:**
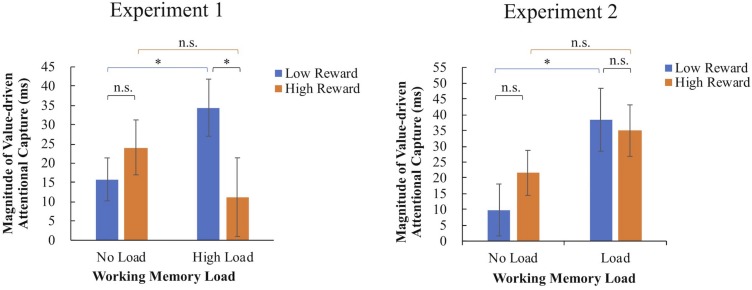
Working memory load interacts with value-driven attention capture. Error bars represent standard error. Fisher’s LSD for reaction time: ^∗^*p* < 0.05, n.s., not significant.

Considering that the meaning of interaction between 2-level working memory load and 3-level reward association might be complicated and indirect, to further explain the interaction, we subtracting RTs of no-reward condition from those of low- and high-reward conditions to get two magnitudes of VDAC, and then reconducted a 2 × 2 repeated measures ANOVA with factors being load and reward (Figure 2, left). Results showed that the interaction between working memory load and reward was significant [*F*(1,17) = 10.52, *p* = 0.005, ${\text{η}}_{\text{p}}^{2}$ = 0.38], whereas neither the main effect of load [*F*(1,17) = 0.13, *p* = 0.72, ${\text{η}}_{\text{p}}^{2}$ = 0.01] or the main effect of reward [*F*(1,17) = 1.40, *p* = 0.254, ${\text{η}}_{\text{p}}^{2}$ = 0.08] was significant. *Post hoc* pairwise comparisons were conducted by using the least significant difference (LSD) method. Attentional capture of low reward was significantly stronger under working memory load condition than under no-load condition (Fisher’s LSD: *p* = 0.016). However, attentional capture of high reward showed no significant difference between load and no-load conditions (Fisher’s LSD: *p* = 0.264). These results indicated that, attentional capture driven by low reward history was strengthened in the dual-task condition while attentional capture of high reward was equivalent between single-task and dual-task conditions. Besides, under no-load condition, there was no significant difference of attentional capture between low- and high-reward conditions (Fisher’s LSD: *p* = 0.309), but under load condition, attentional capture of low reward was greater than that of high reward (Fisher’s LSD: *p* = 0.010).

The 2 × 2 interaction between load and reward (low vs. high reward) above could not fully explain the aforementioned 2(load) × 3(reward) interaction. Considering the data patterns shown in Table 1, it would be helpful to do another 2 × 2 repeated measures ANOVA on no-reward and low-reward conditions (Cohen, 2008). We found a significant interaction between load and reward (no reward vs. low reward) [*F*(1,17) = 7.18, *MS*_e_ = 217.41, *p* = 0.016, ${\text{η}}_{\text{p}}^{2}$ = 0.30], which indicated a steeper increase of RTs with memory load for low-reward condition compared to no-reward condition.

In the digit memory task, a repeated measures ANOVA showed no significant main effect of reward [*F*(2,34) = 0.20, *MS*_e_ = 0.001, *p* = 0.818, ${\text{η}}_{\text{p}}^{2}$ = 0.01] on memory accuracies (92.4, 92.5, and 91.9% in no-reward, low-reward, and high-reward conditions, respectively), meaning that attentional capture by reward association did not impair the performance of memory task.

## Experiment 2

In Experiment 1, we found greater attentional capture driven by low reward history under working memory load, while no significant change of attentional capture caused by high-reward association was observed between load and no-load conditions. One might argue that, in the training or test session, participants might sometimes search for a horizontal or vertical bar instead of specific colors or shapes. To reduce this confounding factor and to verify the results in Experiment 1, we changed all orientations of bars in distractors into axial directions in Experiment 2.

### Participants

Nineteen undergraduates participated in Experiment 2. One participant’s performance in the training session was poor (accuracy = 0.66), which might lead to insufficient trials to learn the association between color and reward. So, the data of this participant was excluded from further analysis. Eighteen participants (6 males and 12 females) were 18–23 years old, with a mean age of 19.94 years old (*SD* = 1.35). All of them had normal or corrected-to-normal visual acuity and had no mental disease history. The experiment was approved by the Ethics Committee of the Department of Psychology, Sun Yat-sen University. Before the experiment, each participant signed informed consent in accordance with the Declaration of Helsinki.

### Materials and Methods

The design, procedure, and most of the stimuli of Experiment 2 were the same as Experiment 1. The only difference was that the bar orientations inside distractors were horizontal or vertical in both the training and test sessions.

### Results

#### Value-Driven Attentional Capture

To ensure the training effect of reward associations, we divided the training session into two phases as in Experiment 1and did a repeated measures ANOVA on RTs. Similar to the results of Experiment 1, we found a significant main effect of training [*F*(1, 17) = 7.91, *MS*_e_ = 421.293, *p* = 0.012, ${\text{η}}_{\text{p}}^{2}$ = 0.32], but no significant effect of reward [*F*(1, 17) = 1.27, *MS*_e_ = 370.26, *p* = 0.28, ${\text{η}}_{\text{p}}^{2}$ = 0.07]. Different from Experiment 1, a significant interaction between training phase and reward [*F*(1, 17) = 5.627, *MS*_e_ = 146.13, *p* = 0.03, ${\text{η}}_{\text{p}}^{2}$ = 0.25] was found, which resulted from a greater RT decrease with training for the high-reward condition (first phase: 563.52 ± 28.42 ms; second phase: 543.16 ± 29.83 ms) compared to the low-reward condition (first phase: 561.88 ± 31.18 ms; second phase: 555.03 ± 35.98 ms).

RTs and error rates of visual search task in the test session are shown in Table 2. A repeated measures ANOVA on RTs showed a significant load effect [*F*(1,17) = 97.64, *MS*_e_ = 3791.33, *p* < 0.001, ${\text{η}}_{\text{p}}^{2}$ = 0.85], a significant reward effect [*F*(2,34) = 13.13, *MS*_e_ = 639.96, *p* < 0.001, ${\text{η}}_{\text{p}}^{2}$ = 0.44], and a marginally significant interaction between them [*F*(2,32) = 2.77, *MS*_e_ = 662.64, *p* = 0.077, ${\text{η}}_{\text{p}}^{2}$ = 0.14]. Reward effects were significant under both no-load condition [*F*(2,34) = 4.00, *MS*_e_ = 526.21, *p* = 0.028, ${\text{η}}_{\text{p}}^{2}$ = 0.19] and load condition [*F*(2,34) = 10.48, *MS*_e_ = 776.39, *p* < 0.001, ${\text{η}}_{\text{p}}^{2}$ = 0.38]. In single visual search task (no-load condition), consistent with previous studies on VDAC and Experiment 1, we also found a significant linear trend of RTs under three value levels (no vs. low vs. high) [*F*(1,17) = 9.15, *MS*_e_ = 4199.89, *p* = 0.008, ${\text{η}}_{\text{p}}^{2}$ = 0.35]. *Post hoc* comparisons showed longer RTs under high-reward condition compared to no-reward condition (Fisher’s LSD: *p* = 0.008) and there was no significant difference between no-reward and low-reward conditions (Fisher’s LSD: *p* = 0.247) or between low- and high-reward conditions (Fisher’s LSD: *p* = 0.138). So, for no working memory load condition, both experiments indicated a linear increment of attentional capture with increased value of reward. As for load condition, *post hoc* comparisons showed significant longer RTs under low- and high-reward conditions compared to no-reward condition (Fisher’s LSD: *p* = 0.001 and *p* < 0.001, respectively), but no significant difference was found between low- and high-reward conditions (Fisher’s LSD: *p* = 0.725).

**TABLE 2:** Descriptive statistics of Experiment 2 (*N* = 18).

	**Reaction time (standard deviation) (in millisecond)**		**Error rate (standard deviation) (in percent)**	
**Distractor type**	**No load**	**Load**	**No load**	**Load**
No reward	807.99 (92.43)	911.10 (89.62)	15.45 (7.02)	14.46 (6.22)
Low reward	817.84 (101.38)	949.52 (92.46)	17.60 (8.36)	16.29 (7.22)
High reward	829.59 (103.74)	946.08 (89.14)	18.14 (7.54)	14.78 (7.01)

A repeated measures ANOVA on error rates showed no significant main effect of reward [*F*(2, 34) = 2.28, *MS*_e_ = 27.62, *p* = 0.118, ${\text{η}}_{\text{p}}^{2}$ = 0.12], no significant main effect of working memory load [*F*(1, 17) = 0.93, *MS*_e_ = 70.46, *p* = 0.349, ${\text{η}}_{\text{p}}^{2}$ = 0.05], and no significant interaction [*F*(2, 34) = 1.16, *MS*_e_ = 22.42, *p* = 0.326, ${\text{η}}_{\text{p}}^{2}$ = 0.06]. So, there was no trade-off between RTs and error rates.

#### The Interaction Between Reward and Working Memory Load

As in Experiment 1, we also calculated the magnitude of attentional capture by subtracting RTs of no-reward condition from those of low- and high-reward conditions and did a 2 × 2 repeated measures ANOVA (Figure 2, right). The main effect of load is marginally significant [*F*(1, 17) = 3.99, *MS*_e_ = 1984.05, *p* = 0.062, ${\text{η}}_{\text{p}}^{2}$ = 0.19], but there was no significant main effect of reward [*F*(1, 17) = 0.455, *MS*_e_ = 682.78, *p* = 0.509, ${\text{η}}_{\text{p}}^{2}$ = 0.03] or significant interaction [*F*(1, 17) = 10.52, *MS*_e_ = 663.92, *p* = 0.228, ${\text{η}}_{\text{p}}^{2}$ = 0.08]. Although the interaction between reward and working memory load was not significant, which is different with Experiment 1, we ran *post hoc* comparisons to make sure that this inconsistency resulted from the high-reward condition. *Post hoc* pairwise comparisons were conducted by using the LSD method. Consistent with findings in Experiment 1, attentional capture of low reward was significantly stronger under working memory load than under no-load condition (Fisher’s LSD: *p* = 0.022), whereas attentional capture of high reward showed no significant difference between load and no-load conditions (Fisher’s LSD: *p* = 0.313). Meantime, no significant difference of attentional capture was found between low and high rewards under either load or no-load condition (Fisher’s LSD: *p* = 0.725 and *p* = 0.138, respectively). Compared with the results in Experiment 1, the difference of interaction between the two experiments appeared to stem from the different patterns in high-reward condition.

To better explain the marginally significant 2 × 3 interaction and data patterns in Table 2, we also did a 2 × 2 repeated measures ANOVA on load and reward (no reward vs. low reward) as in Experiment 1. Again, a significant interaction was found [*F*(1,17) = 6.36, *MS*_e_ = 577.40, *p* = 0.022, ${\text{η}}_{\text{p}}^{2}$ = 0.27], which indicated a steeper RT increase with memory load for low-reward condition compared to no-reward condition. So, combining the results of two separate ANOVAs, the interaction between load and reward could be accounted for by enhanced attentional capture of low reward under working memory load.

## Discussion

Reward history can guide and attract our attention no matter what our current goal is. It is especially essential to know how attention is allocated when people are under a load of a dual task. Under certain conditions, working memory load can improve (SanMiguel et al., 2008) or impair (De Fockert et al., 2001; Lavie and De Fockert, 2005) attentional selection. The current study focused on the interaction between working memory load and VDAC. Both experiments revealed that working memory load enhanced the attentional capture of low reward history but had mild or no effect on the capture of high reward history. Our results suggest that working memory load may not only impair the maintenance of target information but also modulate learned color-reward association.

### Mechanism of Working Memory Load on Value-Driven Attentional Capture

One robust finding in both Experiment 1 and 2 is the enlargement of attentional capture of low reward history under memory load condition relative to no-load condition, which is consistent with what load theory predicts. According to the load theory (Lavie et al., 2004), when the task is relatively easy and perceptual load is low, the remaining cognitive sources will be distributed to irrelevant distractors, and attention is in charge of eliminating them. The efficiency of the elimination process is influenced by working memory (Lavie et al., 2004), and higher working memory load would cause stronger distraction (Muller-Gass and Schröger, 2007; Lavie, 2010). For example, in a visual search task where participants needed to find a unique shape while a unique color appeared as a distractor, memorizing digits at the same time made RTs much longer than no memory task condition (Lavie and De Fockert, 2005). Working memory load will enhance the processing of distractor (De Fockert et al., 2001), which can even improve the processing of a low-contrast Gabor stimulus in the presence of collinear flanking Gabors (De Fockert and Leiser, 2014). In our study, working memory load may hamper the inhibition of learned color-reward association, which enlarged the extent of attentional capture of reward and therefore prolonged the RT to find the target.

Another possible mechanism of memory load effect on attention is that the distribution of attention is dispersive at the beginning of each trial (Eriksen and James, 1986), and it is hard to get focus due to working memory load and insufficient resources (Ahmed and De Fockert, 2012a). Ahmed and De Fockert (2012b) found improved performance during attention to a global level under working memory load. The spread of attention caused by working memory load can even reduce the inattentional blindness (De Fockert and Bremner, 2011).

In Experiment 1, the attentional capture of low reward history was greater than that of high reward history under working memory load condition. To further examine this finding, we considered a possible reason and designed Experiment 2 to test it. We speculated that in Experiment 1, the window of attention could be large and dispersive, and hence participants might in some trials search for the horizontal or vertical bars by only attending their orientations while ignoring the colors or shapes, which made the color-reward associations hard to be built and therefore less capable of capturing attention. In Experiment 2, we made color/shape the only feature defining the target in the training/test session by changing all distracting bars into axial directions (vertical or horizontal). Different from the result of more attentional capture of low reward than high reward under load condition in Experiment 1, in Experiment 2, no significant difference of attentional capture was found between low- and high-reward conditions under load condition. This inconsistent patterns between experiments indicated that high-reward association in Experiment 2 exhibited higher attentional priority compared to Experiment 1, supporting that the manipulation of changing orientations of distractors is an efficient way to strengthen reward associations. Another difference between the two experiments was the training effect of reward association. In the training session of Experiment 1, there was no direct evidence supporting that low and high rewards improved visual search performance in different degrees. However, in the training session of Experiment 2, we found a greater decrease of RT for high-reward condition compared to low-reward condition, which indicated a better training effect of color-reward associations in Experiment 2 than in Experiment 1. Taken together, the inconsistent data patterns of low and high rewards under load condition in two experiments might be due to different strengths of the high-reward association in two experiments. This also implies that association strength during training is a key experimental factor to induce an attentional capture of reward and should be taken carefully.

As for why the load effect on high reward attentional capture is not significant in both Experiment 1 and 2, there might be three possible reasons. The first is the training of reward association might be not enough, which made both attentional capture by reward history and memory load effect hard to be observed. For example, the training session might be a little short, or the associations between reward and color in the training session are relatively weak. Actually, in Experiment 1, participants could finish the training session quite well even when ignoring the colors of shapes. After changing the association strength by making color (red and green) the only feature bound with the target (or reward) in training session in Experiment 2, the mean magnitude of attentional capture by high reward increased with load numerically (although not significant). The second speculation is that attentional capture of high reward already reached the ceiling, which cannot be further enlarged by working memory load. In Experiment 2, attentional capture of both low and high rewards increased with load numerically, and besides, under the load condition, attentional capture of low and high rewards reached a same level. So the results can be a combination of load effect and ceiling effect. Finally, there is a third possibility that working memory load may impair the high-reward association with color held in working memory more than low-reward association, which is a counterforce to capture enlargement caused by less inhibition. Although there is no direct evidence supporting this hypothesis, as we will discuss in the next session in detail, there do exist some findings indicating that working memory load can interfere with reward association stored in memory space.

### Working Memory and Reward Association

In the present study, we did not find a significant difference in attentional capture of high reward between no-load and load conditions. This may be because the reward association is held in working memory, and working memory load may constrain or even impair this information, which makes the facilitation of reward association on distractor processing constant or even reduced. Infanti et al. (2015) found learned feature-reward associations would interfere with mnemonic representations during encoding and holding periods of working memory when no reward was provided. This indicates that reward associations learned through training can be easily invoked by working memory and interact with contents in working memory when the reward-related feature appears. So, it is reasonable that in current experiments, when red and green colors appeared in the test session, the color-reward associations were invoked, and the priority of target information was hampered, leading to attentional distraction. As for load condition, working memory load would occupy memory space, which might interfere with the color-reward association that also stored in memory space. Therefore, the final consequence depended on the level of reward association remained in memory space.

Besides, Gong and Li (2014) found working memory performance was improved when the items were in the high reward-associated color than those in the low reward-associated or non-rewarded color. Their results also showed interaction between reward association and working memory contents. Different from Gong and Li’s (2014) study which focused on how the reward association influences working memory, our study addressed how working memory modulates attention allocation with reward association and added some new information to this field.

In the current study, we used no memory load rather than low memory load to shorten the duration of test session, which could avoid possible attenuation of learned reward associations along with time. A similar design (no load vs. high load) was also adopted in a previous attentional capture study (Lavie and De Fockert, 2005; Experiment 1). A limitation of such designs is that the difference between no-load condition and high-load condition would inevitably include task switching in addition to memory load, which makes task switching a potential confounding factor. Future studies are needed to investigate whether task switching and memory load have different influence on attentional distraction of reward history.

In summary, consistent results were found in two experiments that attentional capture of low-reward association was enhanced under memory load condition relative to no-load condition, while no significant memory load effect was found in attentional capture of high-reward association. We propose that working memory load, which occupies part of cognitive resources, hampers the priority of target information during the process of attentional selection. Our findings extend the knowledge of the influence of working memory load on attentional capture of reward, suggesting that attentional distraction caused by reward association is dynamic and could be modulated by cognitive control.

## Data Availability Statement

The raw data supporting the conclusions of this article will be made available by the authors, without undue reservation, to any qualified researcher.

## Ethics Statement

The studies involving human participants were reviewed and approved by the Ethics Committee of the Department of Psychology, Sun Yat-sen University. Written informed consent to participate in this study was provided by the participants’ legal guardian/next of kin.

## Author Contributions

YW and ZQ designed the research. YW performed the research and analyzed the data. YW, TL, and ZQ wrote the manuscript.

## Conflict of Interest

The authors declare that the research was conducted in the absence of any commercial or financial relationships that could be construed as a potential conflict of interest.

## References

[B1] Ahmed L. De Fockert J. W. (2012a). Focusing on attention: the effects of working memory capacity and load on selective attention. *PLoS One* 7:e4310. 10.1371/journal.pone.0043101 22952636 PMC3429456

[B2] Ahmed L. De Fockert J. W. (2012b). Working memory load can both improve and impair selective attention: evidence from the navon paradigm. *Atten. Percept. Psychophys.* 74 1397–1405. 10.3758/s13414-012-0357-1 22872549

[B3] Anderson B. A. (2015). Value-driven attentional priority is context specific. *Psychon. Bull. Rev.* 22 750–756. 10.3758/s13423-014-0724-0 25199468 PMC4362886

[B4] Anderson B. A. Laurent P. A. Yantis S. (2011a). Learned value magnifies salience-based attentional capture. *PLoS One* 6:e27926. 10.1371/journal.pone.0027926 22132170 PMC3221688

[B5] Anderson B. A. Laurent P. A. Yantis S. (2011b). Value-driven attentional capture. *Proc. Natl. Acad. Sci. U.S.A.* 108 10367–10371. 10.1073/pnas.1104047108 21646524 PMC3121816

[B6] Anderson B. A. Yantis S. (2012). Value-driven attentional and oculomotor capture during goal-directed, unconstrained viewing. *Atten. Percept. Psychophys.* 74 1644–1653. 10.3758/s13414-012-0348-2 22810561 PMC3499680

[B7] Awh E. Belopolsky A. V. Theeuwes J. (2012). Top-down versus bottom-up attentional control: a failed theoretical dichotomy. *Trends Cogn. Sci.* 16 437–443. 10.1016/j.tics.2012.06.010 22795563 PMC3426354

[B8] Baddeley A. (1996). The fractionation of working memory. *Proc. Natl. Acad. Sci. U.S.A.* 93 13468–13472. 10.1073/pnas.93.24.13468 8942958 PMC33632

[B9] Chelazzi L. Perlato A. Santandrea E. Della Libera C. (2013). Rewards teach visual selective attention. *Vis. Res.* 85 58–72. 10.1016/j.visres.2012.12.005 23262054

[B10] Cohen B. H. (2008). *Explaining Psychological Statistics.* Hoboken, NJ: John Wiley & Sons.

[B11] De Fockert J. W. Bremner A. J. (2011). Release of inattentional blindness by high working memory load: elucidating the relationship between working memory and selective attention. *Cognition* 121 400–408. 10.1016/j.cognition.2011.08.01632 21937032

[B12] De Fockert J. W. Leiser J. (2014). Better target detection in the presence of collinear flankers under high working memory load. *Front. Hum. Neurosci.* 8:821. 10.3389/fnhum.2014.00821 25352803 PMC4196630

[B13] De Fockert J. W. Rees G. Frith C. D. Lavie N. (2001). The role of working memory in visual selective attention. *Science* 291 1803–1806. 10.1126/science.1056496 11230699

[B14] Desimone R. Duncan J. (1995). Neural mechanisms of selective visual attention. *Annu. Rev. Neurosci.* 18 193–222. 10.1146/annurev.ne.18.030195.001205 7605061

[B15] Eriksen C. W. James J. D. S. (1986). Visual attention within and around the field of focal attention: a zoom lens model. *Percept. Psychophys.* 40 225–240. 10.3758/BF03211502 3786090

[B16] Failing M. F. Theeuwes J. (2014). Exogenous visual orienting by reward. *J. Vis.* 14 6–6. 10.1167/14.5.6 24819737

[B17] Gong M. Li S. (2014). Learned reward association improves visual working memory. *J. Exp. Psychol. Hum. Percept. Perform.* 40 841–856. 10.1037/a0035131 24392741

[B18] Hickey C. Chelazzi L. Theeuwes J. (2010). Reward guides vision when it’s your thing: trait reward-seeking in reward-mediated visual priming. *PLoS One* 5:e14087. 10.1371/journal.pone.0014087 21124893 PMC2990710

[B19] Infanti E. Hickey C. Turatto M. (2015). Reward associations impact both iconic and visual working memory. *Vis. Res.* 107 22–29. 10.1016/j.visres.2014.11.008 25481632

[B20] Lavie N. (2010). Attention, distraction, and cognitive control under load. *Curr. Dir. Psychol. Sci.* 19 143–148. 10.1177/0963721410370295 26728138

[B21] Lavie N. De Fockert J. (2005). The role of working memory in attentional capture. *Psychon. Bull. Rev.* 12 669–674. 10.3758/BF03196756 16447380

[B22] Lavie N. Hirst A. De Fockert J. W. Viding E. (2004). Load theory of selective attention and cognitive control. *J. Exp. Psychol. Gen.* 133:339. 10.1037/0096-3445.133.3.339 15355143

[B23] Milliken G. A. Johnson D. E. (1984). *Analysis of Messy Data, Volume I: Designed Experiments.* Belmont, CA: Wadsworth. Inc.

[B24] Muller-Gass A. Schröger E. (2007). Perceptual and cognitive task difficulty has differential effects on auditory distraction. *Brain Res.* 1136 169–177. 10.1016/j.brainres.2006.12.020 17223092

[B25] Pashler H. (1994). Dual-task interference in simple tasks: data and theory. *Psychol. Bull.* 116 220–244. 10.1037/0033-2909.116.2.220 7972591

[B26] Raymond J. E. O’Brien J. L. (2009). Selective visual attention and motivation the consequences of value learning in an attentional blink task. *Psychol. Sci.* 20 981–988. 10.1111/j.1467-9280.2009.02391.x 19549080

[B27] Sali A. W. Anderson B. A. Yantis S. (2014). The role of reward prediction in the control of attention. *J. Exp. Psychol. Hum. Percept. Perform.* 40 1654–1664. 10.1037/a0037267 24955700 PMC4313538

[B28] SanMiguel I. Corral M. J. Escera C. (2008). When loading working memory reduces distraction: behavioral and electrophysiological evidence from an auditory-visual distraction paradigm. *J. Cogn. Neurosci.* 20 1131–1145. 10.1162/jocn.2008.20078 18284343

[B29] Theeuwes J. (1992). Perceptual selectivity for color and form. *Percept. Psychophys.* 51 599–606. 10.3758/BF03211656 1620571

[B30] Wang L. Yu H. Zhou X. (2013). Interaction between value and perceptual salience in value-driven attentional. *J. Vis.* 13 5. 10.1167/13.3.5 23479463

[B31] Wolfe J. M. Cave K. R. Franzel S. L. (1989). Guided search: an alternative to the feature integration model for visual search. *J. Exp. Psychol. Hum. Percept. Perform.* 15 419–433. 10.1037//0096-1523.15.3.419 2527952

